# Unique antitumor property of the Mg-Ca-Sr alloys with addition of Zn

**DOI:** 10.1038/srep21736

**Published:** 2016-02-24

**Authors:** Yuanhao Wu, Guanping He, Yu Zhang, Yang Liu, Mei Li, Xiaolan Wang, Nan Li, Kang Li, Guan Zheng, Yufeng Zheng, Qingshui Yin

**Affiliations:** 1 Center for Biomedical Materials and Tissue Engineering, Academy for Advanced Interdisciplinary Studies, Peking University, Beijing 100871, China; 2 Southern Medical University, Guangzhou 510515, China; 3 Department of Orthopedics, Guangdong Key Lab of Orthopedic Technology and Implant, Guangzhou General Hospital of Guangzhou Military Command, 111 Liuhua Road, Guangzhou, Guangdong 510010, China; 4 Department of Materials Science and Engineering, College of Engineering, Peking University, Beijing 100871, China

## Abstract

In clinical practice, tumor recurrence and metastasis after orthopedic prosthesis implantation is an intensely troublesome matter. Therefore, to develop implant materials with antitumor property is extremely necessary and meaningful. Magnesium (Mg) alloys possess superb biocompatibility, mechanical property and biodegradability in orthopedic applications. However, whether they possess antitumor property had seldom been reported. In recent years, it showed that zinc (Zn) not only promote the osteogenic activity but also exhibit good antitumor property. In our present study, Zn was selected as an alloying element for the Mg-1Ca-0.5Sr alloy to develop a multifunctional material with antitumor property. We investigated the influence of the Mg-1Ca-0.5Sr-xZn (x = 0, 2, 4, 6 wt%) alloys extracts on the proliferation rate, cell apoptosis, migration and invasion of the U2OS cell line. Our results show that Zn containing Mg alloys extracts inhibit the cell proliferation by alteration the cell cycle and inducing cell apoptosis via the activation of the mitochondria pathway. The cell migration and invasion property were also suppressed by the activation of MAPK (mitogen-activated protein kinase) pathway. Our work suggests that the Mg-1Ca-0.5Sr-6Zn alloy is expected to be a promising orthopedic implant in osteosarcoma limb-salvage surgery for avoiding tumor recurrence and metastasis.

For the last few decades, bio-inert implants such as pins, screws, or plates made of stainless steels, cobalt-chromium alloys, or titanium alloys are widely accepted for orthopedic applications^1^. However, the permanent metallic internal fixation involves several drawbacks such as stress shielding, chronic inflammation, influence in radiological examinations and a second surgery was necessary for implant removal^1^. Thus, biodegradable biomaterials would be an appropriate solution. In recent years, more and more attentions are taken to magnesium (Mg) and its alloys for a temporary bone implants due to their excellent biocompatibility, comparable mechanical property with natural bones and biodegradability in orthopedic applications^2^,^3^,^4^. Taking the biocompatibility into consideration, essential metal elements including Ca, Zn, Sr were widely selected as alloying elements for Mg-based alloys^5^,^6^,^7^,^8^. Ca is the most abundant metal element in human body and the formation of calcium phosphates during the degradation process would provide more suitable local environment for bone mineralization^9^. Zn exhibits anti-inflammatory effect and stimulates bone formation *in vitro*^10^. Sr is a promising agent in treatment of osteoporosis, and it can promote osteoblast maturation, diminish bone resorption^11^,^12^. And a number of Mg-based alloys such as Mg-Ca^5^, Mg-Zn^13^, Mg-Sr^14^ based alloys had been reported. Nevertheless, these researches were mainly focused on the formation or the mineralization of new bones adjacent to the implants. While for the clinic orthopedic occasions, both post-surgery infection and metastasis or recurrence of the malignant bony tumor during limb reconstruction after the completely removal of the tumors are still intractable problems^15^,^16^. Unfortunately, only few publications evaluated the anti-infection and anti-tumor property of the Mg alloys. Lock *et al.* reported that Mg-4Y alloy exhibited antibacterial property for resorbable ureteral stent applications^17^. However, Robinson *et al.* reported that the it was not the Mg^2+^ itself but the increase of pH during the degradation of Mg alone suppressed the bacterial growth^18^. While for the antitumor property, Chen reported that the anodic oxidation plus heat treatment pure Mg (99.95%) suppressed breast cancer both *in vitro* and *in vivo*^19^. In current stage, the application of Mg and Mg-based alloys were mainly focused on orthopedic bone fixing and cardiovascular stent applications, thus it is essential to evaluate the anti-bony tumor property of Mg and Mg-based alloys. In our previous study^20^, a series of quaternary Mg-Ca-Sr-Zn alloys were fabricated and the *in vitro* anti-bacterial property was conducted. Besides, in recent years, quit a few articles^21^,^22^ reported that Zn^2+^ selectively induced apoptosis in cancer cells^23^,^24^. Elevation of Zn^2+^ concentration can trigger breakdown of mitochondrial membrane potential, caspase activation and cell apoptosis^25^. Moreover, osteosarcoma (OS) is regarded as the most common primary malignant bone tumor^16^. Thus, in our present work, U2OS cell line was selected and the feasibility of the Mg-Ca-Sr-Zn alloys reported in ref. 20 with different extrusion parameters (extrusion ratio: 16:1, extrusion rate: 4 mm/min) to avoid metastasis and recurrence in application for bone tumor prosthesis was evaluated. We determined the effects of Mg-1Ca-0.5Sr-xZn (x = 0, 2, 4, 6 wt%) alloys on the proliferation, vitality, cell cycle, migration, invasion and apoptosis of U2OS cells and the Ti-6Al-4V alloy was set as control . We also made an attempt to understand the potential mechanisms of Mg alloys extracts in regulating the signaling pathway in U2OS cells. Our *in vitro* result suggests that Mg-1Ca-0.5Sr-6Zn would be an optimal bone implant with antitumor property and reduce the risk of metastasis and recurrence.

## Result

### pH value characterization of the extracts

**Figure 1:**
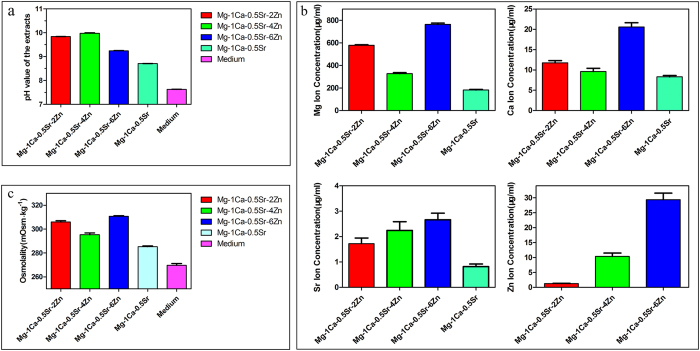
Characterization of the extracts after extracted for 3 days.

The pH value of the alloys extracts was measured with a pH meter (Mettler Toledo, FE20). Figure 1a depicts the pH value of the extracts after extracted in culture medium for 3 days. It could be seen that the pH value of the alloys extracts is higher than the control. And the Zn containing quaternary alloys exhibited a higher pH value than the ternary Mg-1Ca-0.5Sr alloy. While for the quaternary alloys, Mg-1Ca-0.5Sr-6Zn alloy extract possessed the lowest pH value.

### Ion concentration of the extracts

The ion concentration of the extracts was detected by ICP-AES. And the concentration of the Mg, Ca, Sr, Zn ions in the alloys extracts is illustrated in Fig. 1b. As is shown in Fig. 1b, the concentration of Mg, Ca, Sr ions in the Mg-1Ca-0.5Sr alloy extract were significant lower than the quaternary Zn containing alloys. While for the quaternary alloys, both the concentration of Mg ion and Ca ion were lower for the Mg-1Ca-0.5Sr-4Zn alloy. Mg-1Ca-0.5Sr-6Zn alloy exhibited the highest ion concentration. And the concentration of Sr ion and Zn ion was increased with the increment of the Zn content in the quaternary alloys. In all the alloy extracts groups, Mg ion exhibited significant higher concentration than other three alloying elements. The concentrations of Zn ion in the Mg-1Ca-0.5Sr-2Zn and Mg-1Ca-0.5Sr-4Zn alloys are 1.26 ± 0.11 μg/ml and 2.37 ± 0.13 μg/ml, respectively. While for the Mg-1Ca-0.5Sr-6Zn alloy, the concentration of Zn ion is about 29.35 ± 2.19 μg/ml, which is significantly higher than other two quaternary alloys.

### Osmolality of the extracts

The osmolality of the extracts medium was detected with Micro-Sample Osmometer (Fiske 210) and the result is shown in Fig. 1c. From Fig. 1c, we can find that the osmolality of all the alloys extracts was higher than the control. The ternary Mg-1Ca-0.5Sr alloy extract showed a lower osmolality than Zn containing quaternary alloys extracts. While for the quaternary alloys, Mg-1Ca-0.5Sr-4Zn alloy exhibited the lowest osmolality and Mg-1Ca-0.5Sr-6Zn alloy exhibited the highest osmolality. The addition of Zn in the Mg-1Ca-0.5Sr alloy lead to the increment of the osmolality of the extracts.

### Inhibition of osteosarcoma cell proliferation and vitality

**Figure 2:**
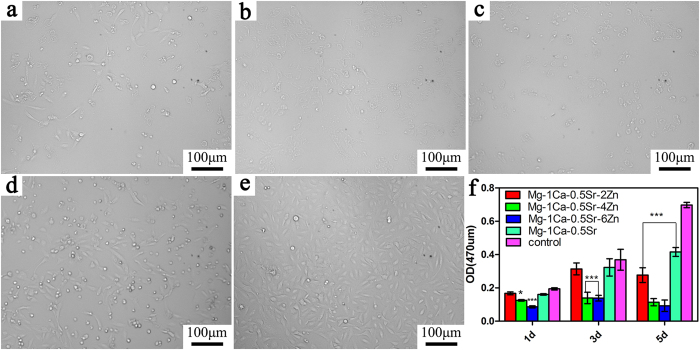
MTT results of the cells cultured in alloy extracts for different days. Cell morphology was observed by inverted phased contrast microscope on day 5: (**a**) Mg-1Ca-0.5Sr-2Zn; (**b**) Mg-1Ca-0.5Sr-4Zn; (**c**) Mg-1Ca-0.5Sr-6Zn; (**d**) Mg-1Ca-0.5Sr; (**e**) Ti-6Al-4V control; (**f**) OD value of the cells cultured in alloy extracts for different days. *compared to the control group (Ti-6Al-4V), One symbol, p < 0.05; Two symbols, p < 0.01, three symbols, p < 0.001.

The proliferation ability and the cell morphology of the U2OS cells cultured in alloys extracts is shown in Fig. 2, and the Ti-6Al-4V alloy was set as control. As we can see from Fig. 2f, the cell proliferation ability after incubated in the alloys extracts exhibited a time-dependent tendency. The OD values in the Mg-1Ca-0.5Sr extract and the control group were increased with the increasing incubation time. While for the alloys with 4 and 6 wt% Zn content alloys, the cell proliferation ability only exhibited little fluctuant during the incubation periods with a relatively lower OD values. Furthermore, the cell viability decreased with the increment of the Zn contents in the alloys. When compared with the Ti-6Al-4V control group, the proliferation rate of the U2OS in Mg-1Ca-0.5Sr-(4, 6 wt%) Zn extracts was significantly lower in day 1 and day 3 (P < 0.05). And in day 5, all the Mg alloys exhibited a significantly lower cell proliferation (P < 0.05). During the whole incubation periods, the Mg-1Ca-0.5Sr and Mg-1Ca-0.5Sr-2Zn alloys exhibited similar cell proliferation ability. As is shown in Fig. 2a–e, we can find that the cell numbers in the view were decreased with the increment of Zn contents in the alloys. The cell numbers in the Mg-1Ca-0.5Sr and Ti-6Al-4V extracts were obviously higher than other alloys.

**Figure 3:**
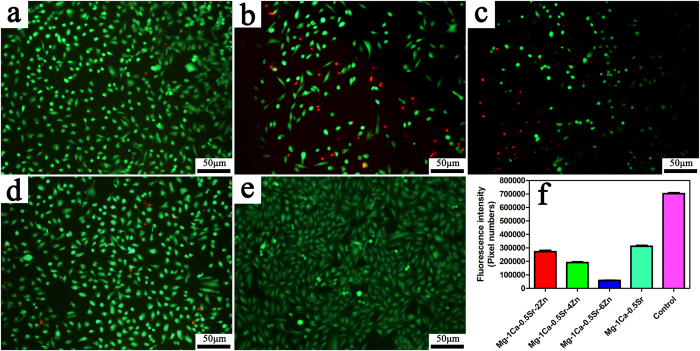
Live/Dead staining of the U2OS cells after incubated in alloy extracts for 5 days, red represent for the dead cells and green for the live cells. (**a**) Mg-1Ca-0.5Sr-2Zn; (**b**) Mg-1Ca-0.5Sr-4Zn; (**c**) Mg-1Ca-0.5Sr-6Zn; (**d**) Mg-1Ca-0.5Sr; (**e**) Ti-6Al-4V control; (**f**) quantitative analysis of fluorescent intensity the live cells in alloys extracts.

Figure 3 presented the Live/Dead staining fluorescence images of the U2OS cells after incubated in the alloys extracts for 5 days. For quantitative analysis (Fig. 3f), when compared to the control group, all of the Mg alloys groups demonstrate a reduction in cell number but most of the cells were alive (in green) in Mg-1Ca-0.5Sr-2Zn, Mg-1Ca-0.5Sr alloys extracts. At the same time, more dead cells (in red) can be seen in the Mg-1Ca-0.5Sr-4Zn and Mg-1Ca-0.5Sr-6Zn alloys group. The cells cultured in the Zn containing quaternary alloys exhibited an unhealthy shrinkage shape (Fig. 3a–c). The florescence staining test results were in accordance with the MTT assays, higher Zn content in the alloys exhibited higher inhibition efficiency. In terms of the proliferation and vitality, alloys with higher Zn content exhibited better suppression efficiency.

### Suppression of U2OS cells migration and invasion

**Figure 4:**
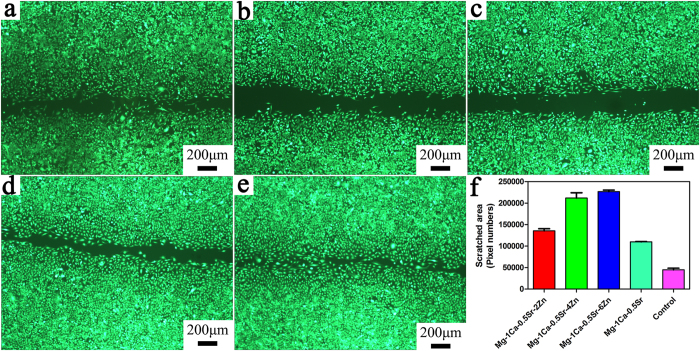
The effect of different alloys extracts on the migration ability of U2OS cells. (**a**) Mg-1Ca-0.5Sr-2Zn; (**b**) Mg-1Ca-0.5Sr-4Zn; (**c**); Mg-1Ca-0.5Sr-6Zn; (**d**) Mg-1Ca-0.5Sr; (**e**) Ti-6Al-4V control; (**f**) denuded areas of the scratched gaps after incubated in the extract for 24 h. *compared to the control group (Ti-6Al-4V), three symbols, p < 0.001.

Wound healing scratch assay was performed in order to evaluate whether the alloys extracts played a role in inhibiting the migration of the U2OS cells. After 24 h incubation with the alloys extracts, the migration of the cells along the scratched section was measured. It can be observed in Fig. 4 that the cell migration rate was varied with different alloys extracts. In Mg-1Ca-0.5Sr-(2, 4, 6 wt%)Zn alloys extracts, the cells numbers in the denuded area were lower than in the Mg-1Ca-0.5Sr and Ti-6Al-4V alloys extracts. The distance between the scratched gaps increased with the increasing Zn content in the quaternary alloys. After 24 h, the scratched areas were almost closed in the control group. For quantitative analysis, Fig. 4f depicts the denuded area after incubated with the alloys extracts. As we can see from Fig. 4f the denuded areas increased with the increment of the Zn contents. And the denuded area was lower in the Mg-1Ca-0.5Sr and Ti-6Al-4V control group, indicating that the cells in the control group exhibited faster migration rate.

**Figure 5:**
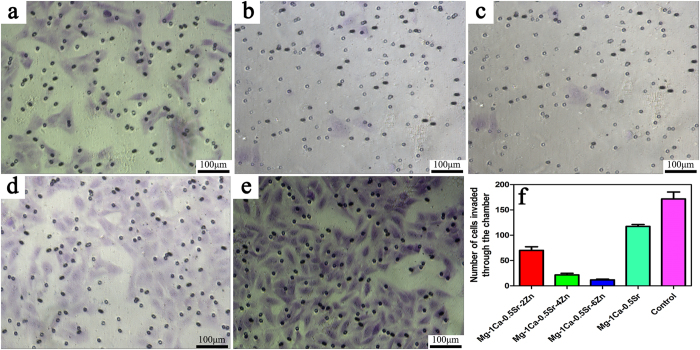
The effect of different alloys extracts on the invasion ability of U2)S cells, (**a**) Mg-1Ca-0.5Sr-2Zn; (**b**) Mg-1Ca-0.5Sr-4Zn; (**c**) Mg-1Ca-0.5Sr-6Zn; (**d**) Mg-1Ca-0.5Sr; (**e**) Ti-6Al-4V control; (**f**) numbers of cells invaded through the chamber.

The effects on the cell invasion were evaluated by 24-well plate inserts with Matrigel coated chambers. The microscopic photograph after crystal violet staining and the cell numbers invaded through the chambers are shown in Fig. 5. As we can see from Fig. 5f, the number of cells invaded through the Matrigel coated chambers were lower in Mg-1Ca-0.5Sr-(2, 4, 6 wt%)Zn alloys extracts than the Mg-1Ca-0.5Sr and control group. While for the Mg-1Ca-0.5Sr and control group, the invaded cell number was significantly higher than Zn containing alloy group. And alloys with higher Zn content resulted in fewer cells invaded through the chambers. Furthermore, only few cells can be seen in the Mg-1Ca-0.5Sr-6Zn alloy extract group, indicating that the invasion ability was almost completely suppressed by the alloy extract. These results suggest that Mg-1Ca-0.5Sr-6Zn alloy process better effect on suppressing the invasion of U2OS cells *in vitro*.

### Induced U2OS cells cycle arrest and cell apoptosis

**Figure 6:**
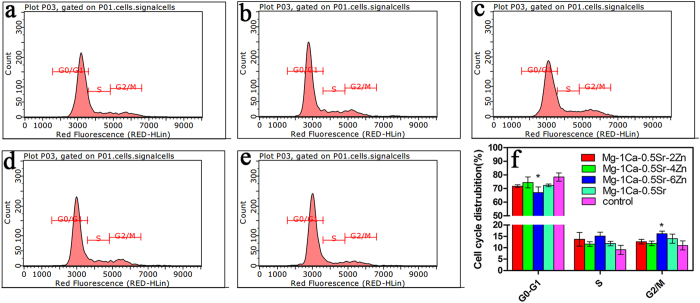
Roles of different alloys extracts in cell cycle of U2OS cells. (**a**) Mg-1Ca-0.5Sr-2Zn; (**b**) Mg-1Ca-0.5Sr-4Zn; (**c**) Mg -1Ca-0.5Sr-6Zn; (**d**) Mg-1Ca-0.5Sr; (**e**) Ti-6Al-4V; (**f**) quantitative analysis of the cell cycle distribution. *compared to the control group (Ti-6Al-4V), One symbol, p < 0.05.

To explore the potential mechanism of suppressing cell growth by the Mg alloys extracts, cell cycle distribution analysis by flow cytometry was performed. Figure 6 illustrates the flow cytometry data of the cell cycles analysis in U2OS cells. The percentage of cells in G0/G1, S and G2/M phases of the cell cycle were calculated. As is illustrated in Fig. 6, we can find that two days’ incubation in the five alloys extracts induced differential changes in the cell cycle distribution of the U2OS cells. An accumulation of cells in G2/M phase that accompanied by a decrease of cells with G0/G1 phase was observed in U2OS cells for the Mg alloys extracts groups. Among the quaternary alloys extracts group, Mg-1Ca-0.5Sr-6Zn alloy exhibited fewer cells in G0/G1 phase and more cells in S phase and G2/M phase. Cells numbers in G2/M and G0/G1 phases after incubated in Mg-1Ca-0.5Sr-6Zn alloy extracts exhibited a significant difference when compared with the control group(P < 0.05). This result indicated that the magnesium alloys extracts inhibited U2OS cell proliferation via inducing G2/M phase arrest and G0/G1 phase reduction.

**Figure 7:**
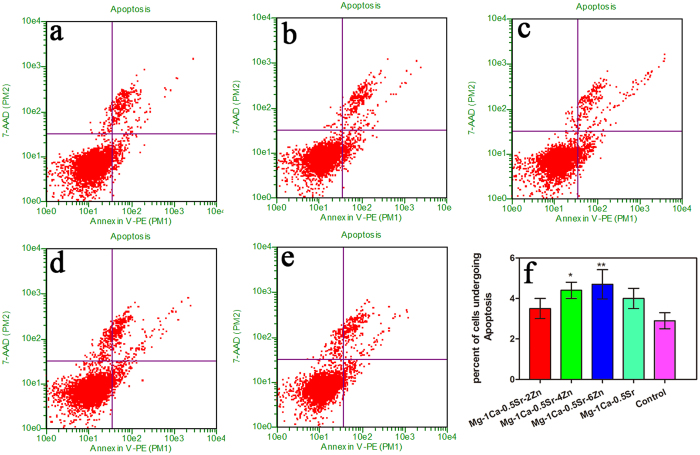
Roles of different alloys extracts in cell apoptosis of U2OS cells. (**a**) Mg-1Ca-0.5Sr-2Zn; (**b**) Mg-1Ca-0.5Sr-4Zn; (**c**) Mg-1Ca-0.5Sr-6Zn; (**d**) Mg-1Ca-0.5Sr; (**e**) Ti-6Al-4V; (**f**) apoptosis rate of the cells after incubated in the extracts. *compared to the control group; One symbol, p < 0.05; Two symbols, p < 0.01.

Apoptosis plays a vital role in the programed cell death. To elucidate the apoptotic potential of the alloys extracts on U2OS cells, induction of apoptosis was investigated by flow cytometry analysis. As is shown in Fig. 7, cells cultured with Mg alloys extracts displayed higher early apoptosis rate. The percentages of the apoptotic cells for the quaternary alloys were increased with the increment of the Zn content in the alloys. The percentage of apoptotic cells in Mg-1Ca-0.5Sr-4Zn and Mg-1Ca-0.5Sr-6Zn alloys were 4.4% and 4.5%, respectively. And these two alloys exhibited significantly higher cell apoptosis rate when compared with the Ti-6Al-4V control group (P < 0.05). In addition, despite the fact that there was no statistical difference between Mg-1Ca-0.5Sr-(0, 2 wt%) Zn alloys groups and the control group in terms of the number of cells undergoing apoptosis, they also manifest an inhibition effect to the U2OS cells. The result suggest that the Mg alloys showed an apoptosis effect to the U2OS cells, especially for the Mg-1Ca-0.5Sr-6Zn.

### Protein expression level in MAPK pathway

**Figure 8:**
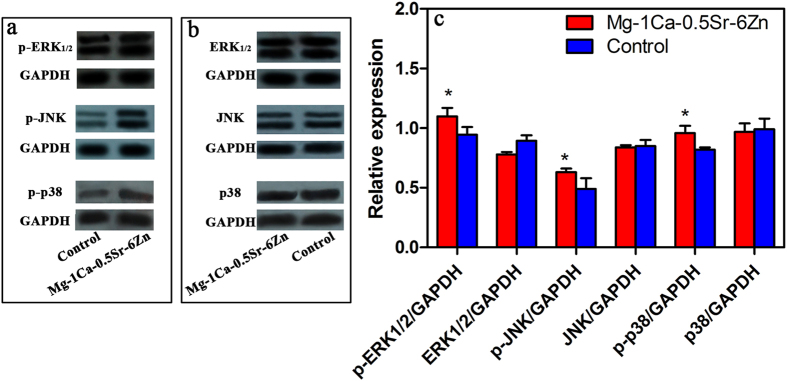
The expression of target proteins involved in MAPK pathway. (**a,b**) protein expression; (**c**) quantitative analysis of the relative expressed proteins. *compared to the control group (p < 0.05).

According to our previous studies, the Mg-1Ca-0.5Sr-6Zn alloy exhibited a better *in vitro* antitumor property. We further evaluated the protein expression level of the MAPK pathway in the U2OS cells after cultured in the Mg-1Ca-0.5Sr-6Zn extracts. the expression level of p38, JNK and ERK1/2 proteins and phosphorylate p-p38, p-JNK, p-ERK1/2, which were involved in MAPK pathway, is shown in Fig. 8. The expression level of p38, JNK, ERK1/2 is lower than the control. While the phosphorylated proteins expressed in the Mg-1Ca-0.5Sr-6Zn alloys extracts group are significant higher than the control group, indicating that p-p38, p-ERK1/2 and p-JNK were all activated.

### Protein expression level in mitochondria pathway

**Figure 9:**
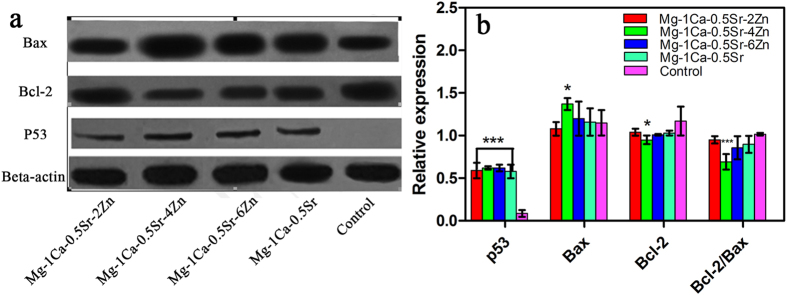
The expression of target proteins involved in mitochondria pathway. (**a**) protein expression; (**b**) quantitative analysis of the relative expressed proteins. *compared to the control group. One symbol, p < 0.05; three symbols, p < 0.001.

As a major checkpoint of apoptotic regulation, mitochondria play a vital role in the cell apoptosis. The proteins such as p53, Bax and Bcl-2 which involved in the mitochondrion pathway were essential to affect the mitochondrial function and regulate the release of apoptosis related factors. The expression level of the p53, Bax and Bcl-2 proteins in the U2OS cells after cultured with the alloys extracts is illustrated in Fig. 9. As is shown in Fig. 9, the expression level of p53 exhibited an ascending trend with increment Zn content in the alloys and all Mg alloy groups showed a significant difference compared to the control groups. Moreover, the expression of anti-apoptotic protein Bcl-2 was significantly downregulated and the expression of pro-apoptotic protein Bax was significantly upregulated in the alloy extracts in comparison to the control group. Besides, the ratio of Bcl-2/Bax is also lower in the Mg alloys extracts groups.

## Discussion

Accompanied with osteoid formation or osteolytic lesions of bones, osteosarcoma is the most common primary malignant bone tumor characterized by its metastasis and high local recurrence rate, which frequently threatens the health of patients^26^,^27^. In recent years, the combination of aggressive chemotherapy and radical surgical resection treatment of OS improved the 5-year survival rates to about 50–70%^28^,^29^,^30^. Nevertheless, most chemotherapeutics destroy both cancer and healthy cells and resulted in severe side effects that affect the life quality and even shorten the overall survival rate of the patients^31^,^32^. Thus, surgery remains an indispensable part of OS treatment^33^,^34^. For the limb-salvage surgery, in order to extend the lives and improve the life quality of the patients, the tumor must be resected as thoroughly as possible to avoid its metastasis and recurrence. After the completely removal of the tumor, limb reconstruction plays a key role in avoiding postoperative fractures and keeping the patients’ quality of life^35^. Increasing evidences^36^,^37^,^38^ had proved that Zn was closely related with the survival, proliferation, cell cycle arrest, apoptosis and metastasis of tumor cells via regulating different signaling pathways. It had also been reported that Zn inhibited the growth of many tumor cells such as A549 cells, Caco-2 cells, HeLa cells, HepG2 cell, HT29 cell and so on^21^,^36^,^39^. Thereby, we evaluated the antitumor property of the Mg-Ca-Sr-Zn alloys, which had been proved to have antibacterial property *in vitro*^20^. The influence of the Mg-1Ca-0.5Sr-(0, 2, 4, 6wt%) Zn alloys on the viability, cell cycle, apoptosis, cell migration and invasion of the U2OS cells as well as the potential relevant mechanism were also studied.

Our *in vitro* cytotoxicity assay showed that the Zn containing alloys extracts significantly inhibit the growth and viability of the U2OS cells (Figs 2 and 3). And the cells cultured with the quaternary alloys extracts were in unhealthy shrinkage shape (Fig. 2a–c). The inhibition efficiency was in a dose-dependent manner, higher Zn contents in the alloys lead to a stronger inhibition effect. As we know, cell cycle plays a vital role in cells proliferation, division and duplication. There are five distinct phases in the cell cycle: G0 (quiescence), G1 (cell size increasing), S (DNA synthesis), G2 (continued cell growth), M (mitosis and cytokinesis). During the proliferation procedure, cell cycle control is ordered. The progress of cell cycle is normally monitored by different checkpoints in G1/S intra-S and G2/M. These checkpoints control the progression through the phases of the cell cycle. Moreover, the G2/M transition is one of the two main checkpoints that used by the cell to regulate the progression of the cell cycle. The cell cycle analysis data demonstrated that the percentage of G2/M phase in U2OS cells increased after treated with Mg-1Ca-0.5Sr-(0, 2, 4, 6) Zn extracts (Fig. 6, P < 0.01 vs. control) and the percentage of G0/G1 phase cells showed an opposite trend. These results suggest that the Mg alloys extracts suppressed U2OS cell growth by inducing G0/G1 phase reduction and G2/M phase arrest.

The ability of invasion and metastasis are critical biological characteristics of tumor cells. Despite the fact that advancements of the multimodal treatment had been achieved during the past years. Tumor metastasis is still one of the most difficult problem faced by the surgeon^40^. Thus, to suppress the tumor cells migration and invasion may be one of the keys to prevent tumor metastasis. The lower migration rate of the cells in the Zn containing alloys extracts in the scratch assay (Fig. 4) as well as less invaded cells in the invasion test (Fig. 5) clearly revealed that the Mg alloy extracts inhibited the cell migration, and behaved a dose-depended manner *in vitro*.

Recent studies^40^,^41^ found that the activation of three major proteins of MAPK pathways can obstruct cancer cell migration and invasion. Therefore, to elucidate the role of the signaling pathways that played in the Mg alloys extracts mediated responses in U2OS cells, Mg-1Ca-0.5Sr-6Zn alloy extract was selected to further investigated the effect of the alloy on the activation of MAPK pathway due to its most efficiency inhibition effect on the U2OS cells. We found that the U2OS cells treated with Mg-1Ca-0.5Sr-6Zn alloys extracts showed a significantly higher expression level of the phosphorylation of p-p38, p-JNK and p-ERK1/2 (Fig. 8). Therefore, we speculate that the alloys extracts can effectively prevent the metastasis of U2OS cells mainly by activating MAPK pathways.

Apoptosis is a common form of programmed cell death and one of the basic characteristics of cancer cells is to avoid apoptosis and continue to propagate. Thus, to investigate the apoptotic effect of the Mg alloys extracts on U2OS cells, cell apoptosis was examined and western blotting analysis was also employed to determine the expression of apoptosis-related proteins. As the powerhouses of the cells, the mitochondria have been proposed as a potential drug target for cancer therapy via the permeabilization of the mitochondrial outer membrane of the mitochondria-mediated apoptotic pathway^42^. As we all known, the interaction of Bcl-2 family proteins played an important role in this step, which consist of pro-apoptotic protein Bax as well as competing anti-apoptotic protein Bcl-2, and the balance between these two groups proteins determined the fate of cells^43^. In the present study, we observed that the alloys extracts can significantly down-regulated the level of Bcl-2 protein and up-regulated the levels of Bax (Fig. 9). Besides, the result also indicated that the imbalance ratio of Bcl-2/Bax contributed to the disruption of mitochondrial potential, suggesting that the involvement of an intrinsic apoptotic pathway in U2OS cells treated by the Mg alloys extracts. Although, the proliferation, viability assay, scratch assay as well as invasion assay released that Mg-1Ca-0.5Sr-6Zn alloy showed better antitumor property *in vitro*. However, our results showed that the cells after treated with Mg-1Ca-0.5Sr-6Zn extracts, the expression level of Bax was lower while the expression level of Bcl-2 was higher when compared with the cells treated with Mg-1Ca-0.5Sr-4Zn extracts (Fig. 4). As we can see from Figs 2 and 3, the cells in Mg-1Ca-0.5Sr-6Zn extracts exhibited a lower proliferation rate and an unhealthy morphology. The Live/Dead staining assay also released that the amounts of viable cells in Mg-1Ca-0.5Sr-6Zn alloys extracts also lower than other two Zn containing alloys (Fig. 3). Since the Mg-1Ca-0.5Sr-6Zn alloys showed higher toxicity to the U2OS cells, the severe cell damage caused by the Mg-1Ca-0.5Sr-6Zn alloy extracts may attribute to the disorder of the homeostasis of the cells, resulting in severe disturbance of the regulation of the protein synthesis^44^.

In addition, we also adopted the western blotting technique to evaluate the protein expression level of p53, which are regarded as a factor to trigger cell-cycle arrest or apoptosis^45^. Under normal conditions, p53 levels are maintained at a low state by virtue of extremely short half-life of the polypeptide. When encounter DNA damage, expression level of p53 protein dramatically rise within minutes, leading to the activation of a number of genes and resulting in cell-cycle arrest or apoptosis. In our present study, significantly higher expression level of p53 are observed among the group treated with the Mg alloys extracts when compared to the control group, which suggest that the Mg alloys extracts may also result in the death of the U2OS cells through the similar mechanism as the previous studies reported.

A suitable culture medium condition is essential for the growth of cells. As biodegradable materials, the gradually degradation of Mg-based metals in the extracts medium resulted in the releasing of metal ions accompanied with higher pH value and osmolality in the surrounding medium. Thus, these changes in the culture condition may also be a factor that induced apoptosis in mammalian cells^46^. According to ref. 47, the optimal osmolality range for cellular growth is about 240 to 370 mOsm kg^−1^. Fischer *et al.*^48^ reported that osteoblasts after cultured in the extracts medium of pure Mg, Mg-1Ca and Mg-0.6Ca alloys with an osmolality between 347 and 353 mOsm kg^−1^ exhibited high metabolic activity(over 90%). Wang *et al.*^49^ also reported that osteoblasts and MC3T3-E1 cells cultured in the medium with an osmolality below 400 mOsm kg^−1^ exhibited comparable viability when compared to the control group. Higher osmolality (over 450 mOsm kg^−1^) significantly reduced the cell viability. It had been reported^50^ that murine embryonic stem cells (mESC) exhibited the higher growth rate when the osmolality of the culture medium was 300 mOsm kg^−1^ and 350 mOsm kg^−1^. In this work, the osmolality of the alloys extracts ranges from 275.33 ± 1.53 mOsm kg^−1^ to 310.67 ± 0.58 mOsm kg^−1^, which was in the suitable ranges for cell growth (Fig. 1c). Thus, the osmolality of the alloys extracts would not be the matter for the antitumor property of the alloys.

Zn^2+^has been reported as a factor that played a vital role in inducing the cancer cell death^51^,^52^,^53^. The concentration of Zn ion in the alloys extracts was 1.26 ± 0.11 μg/ml for Mg-1Ca-0.5Sr-2Zn alloy, 10.37 ± 1.12 μg/ml for Mg-1Ca-0.5Sr-4Zn alloy and 29.35 ± 2.19 μg/ml for Mg-1Ca-0.5Sr-6Zn alloy (Fig. 1b). Our results clearly showed that the inhibition efficiency of the alloys were proportional to the Zn content in the alloys extracts. Wahab *et al.* reported that after treatment with ZnO nanoparticles at a concentration of 2.5–10 μg/ml, no significant changes in the cell cycles of MCF-7 cells can be found. However, after treatment with 25 μg/ml ZnO nanoparticles, more MCF-7 cells in the G2/M phase were observed. Further increase ZnO nanoparticle concentration lead to a reduction of cells in normal G1 phase^37^. Akhtar *et al.* also reported that 5 μg/ml ZnO nanoparticles did not produce significant reduction in viability of cancer cells (HepG2, A549, and BEAS-2B cells). When the concentration increased to 15 μg/ml, significant reduction in cell viability was observed^38^. Kee *et al.*^54^ concluded that the dissolution of ZnO nanoparticles into ions is a vital step in exhibiting its toxicity effects. 20 μg/ml ZnO nanoparticles lead to robust phosphor-p53 and total p53 levels. With lower Zn ion concentration (below 10 μg/ml), Mg-1Ca-0.5Sr-2Zn alloy exhibited similar reduction rate of cytotoxicity, migration ability, invasion ability and apoptosis of the U2OS cells when compared with the ternary Mg-1Ca-0.5Sr alloy. The highest Zn ion concentration in the Mg-1Ca-0.5Sr-6Zn alloy extracts showed the best antitumor property (Fig. 1b, Figs 2 and 3). However, despite the fact that there was no Zn in the Mg-1Ca-0.5Sr alloy, it also exhibited a certain effect in inhibiting the proliferation of U2OS cells, suggesting that Zn^2+^was not the only factor that affecting the viability of U2OS cells in alloy extracts (Fig. 2). On one hand, the pH value of the Mg-1Ca-0.5Sr alloy extract was about 8.71 ± 0.01, as we can see from Figs 2 and 3, it only exhibited little inhibition effect on the cell viability of U2OS cells. While for the 2Zn group, the pH value of the alloy extract was about 9.84 ± 0.01, and it showed similar cell cytotoxicity with the ternary alloy group according to the MTT assay and live/dead staining assay. On the other hand, the pH value of the quaternary alloys extracts only exhibited a little fluctuation, however, the inhibition efficiency was quite different from each other. The inhibition efficiency was proportional to the Zn^2+^concentration in the extracts. Higher Zn^2+^concentration lead to higher inhibition effect of the U2OS cells. In present study, the pH of extracts (between 8.71 and 9.98) was higher than the control group (Fig. 1a), it may also act as an inhibitor to the cell growth. However, the pH value of the extracts would not be the main factor for the inhibition effect of the alloys extracts, but Zn^2+^ in the extracts played a vital role.

**Figure 10:**
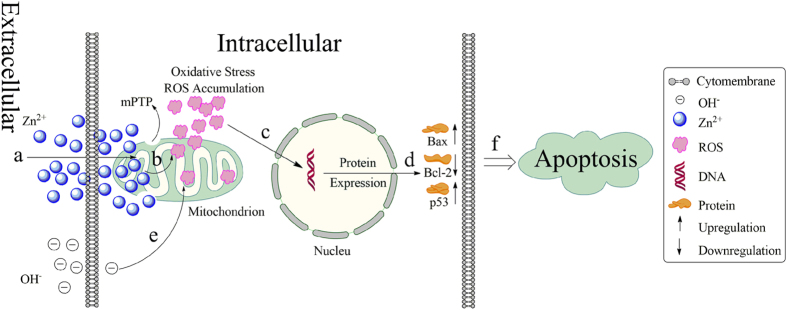
Schematic illustration of possible process of the extracts induced U2OS cell apoptosis. (**a**) transportation of Zn^2+^ into cell and higher intracellular Zn^2+^ concentration induced dysfunction of mitochondrion; (**b**) accumulation of ROS in mitochondrion; (**c**) the oxidative stress regulates the gene expression; (**d**) regulation of protein expression levels; (**e**) extracellular OH^-^ may also induced ROS accumulation; (**f**) the upregulation of Bac and p53 and down regulation of Bcl-2 lead the cell apoptosis.

Based on the previous study, the probably mechanism that the prepared alloys induced cell apoptosis was schematically illustrated in Fig. 10. The degradation of the alloys resulted in the releasing large amounts of Zn^2+^ in the extracellular matrix. The Zn^2+^ was subsequently transported into the cell via the ion channels in the cell membrane. The increasing Zn^2+^ concentration in the cells lead to the dysfunction of mitochondrial, for instance, mitochondrial permeability transition pore (mPTP), loss of the mitochondrial transmembrane electrochemical gradient, respiratory block. As a result, the ROS accumulates in the live cells, and cause cellular oxidative stress and subsequently regulated the expression of the mitochondrial pathway related proteins. The upregulated expression of pro-apoptotic proteins Bax, p53, down regulated anti-apoptotic protein Bcl-2 finally caused the cell apoptosis. The tumor cells were also known for its fast metabolism rate. And the fast metabolism rate resulted in more energy consumption for the tumor cells. As the powerhouse of the cells, the dysfunction of the mitochondrial would significantly suppressed the growth of the tumor cells. While for the normal cells, such as MC3T3-E1 cells, the alloys extracts exhibited negligible cytotoxicity^20^. According to the report^55^, the high pH value also can weaken the ability of cell itself on scavenging free radicals by suppressed the activity of superoxide dismutase(SOD), peroxidase, and catalase in cells which resulted in the excess free radicals accumulated in cells. Thus, it should be pointed that the releasing OH^-^ form the degradation of the alloys may also contribute to the accumulation of the ROS in the live cells to some extent. And recently, some article^44^,^54^,^56^,^57^,^58^ reported that Zn^2+^ releasing from the Mg alloys and the rising pH can trigger excessive ROS, and then corporately lead to the osteosarcoma cell death. The result in our study may suggest there exist a similar mechanism in inhibiting the growth of U2OS, which would be a reasonable explanation for our study.

Taken together, we investigated the antitumor effects of the Mg-1Ca-0.5Sr- based Mg alloys on the human U2OS cells. Based on our present work, the Mg-1Ca-0.5Sr-6Zn alloy extracts significantly reduced the proliferation, viability, migration ability as well as the invasion ability of the U2OS cells *in vitro*. We further found out that the extracts-induced cell apoptosis was mediated by the mitochondrial pathway, and the MAPK signaling pathway play a vital role in inhibiting the metastasis of U2OS cells. Our results suggested that the Mg-1Ca-0.5Sr-6Zn alloy will be a potential candidate for antitumor implant. However, further investigation should be conducted to clarify the detail molecular mechanisms of extracts-induced cell apoptosis and suppression of migration. Although our present work demonstrated that the Mg-1Ca-0.5Sr-6Zn alloy exhibited better antitumor property, however, the *in vivo* tests for the future studies should also be applied to better understand the antitumor behavior of the alloys.

## Conclusions

Our present study demonstrate that the series of Mg alloys extracts inhibits U2OS cell proliferation via cell cycle G2/M arrest and promotes apoptosis via the mitochondrion-dependent pathway. Meanwhile the alloys extracts suppressed the metastasis of the U2OS via MAPK pathway. Mg-1Ca-0.5Sr-6Zn alloy showed the best antitumor property *in vitro*. This study may provide information for the introduction of a promising type of bioimplant prosthesis in limb reconstruction after surgical resection treatment of OS.

## Methods

### Alloys extracts preparation and characterization

RPMI-1640 medium extracts of the alloys for the *in vitro* antitumor assays were prepared as follows. Before the extract procedure, all the alloy samples were sterilized in 70% ethanol for 30 min and rinsed with a 0.85% NaCl solution. Then Mg alloy samples and Ti-6Al-4V alloy were immersed in RPMI-1640 medium (Gibco) with an extraction ratio of 1.25 cm^2^/ml and incubated at 37 °C in a humidified atmosphere of 5% CO_2_ for 72 h. Subsequently, all the alloy extracts were centrifuged at 3000 rpm for 5 min to remove the alloys particulates, the supernates were then collected and 10% fetal bovine serum (FBS) (Gibco) was added. Subsequently, the alloys extracts were stored at 4 °C prior to the experiments. While for cell migration and invasion assays, the alloys extracts without the addition of FBS were used. The pH value, ion concentration and osmolality of the alloys extracts were also characterized.

### Cell culture

The human osteosarcoma cell line U2OS was adapted to evaluate the *in vitro* antitumor property of the prepared alloys. Cells were purchased from the Type Culture Collection of the Chinese Academy of Sciences. Cells were maintained in RPMI-1640 supplemented with 10% FBS in a humidified atmosphere with 5% CO_2_ at 37 °C. The medium was changed every two or three days.

### Cell proliferation assay

U2OS cells were seeded in 96-well plates and incubated with RPMI-1640 supplemented with 10% FBS at a density of 2 × 10^4^ cells/ml in a humidified atmosphere with 5% CO_2_ at 37 °C for 1 day to allow attachment. The medium was then replaced by 100 μl of alloys extracts. At each of the designated time points(Day 1, 3 and 5), the extracts medium was then removed and 200 μL culture medium contains 20 μL of MTT (Sigma, USA) solution (5 mg/ml in PBS) was added, followed by continuous incubation for 4h at 37 ^o^C. After the culture medium was removed, the formazan reaction products were dissolved in 150 μL dimethylsulfoxide (DMSO) for 20 min. The absorbance value at 490 nm was measured in a microplate reader. Each experiment was carried out in triplicate.

### Cell Viability

For viability staining studies, cells were seeded in 24-well plates at a concentration of 1 × 10^5^ cells/ml in a humidified atmosphere at 37 °C with 5% CO_2_ for 1 day to allow attachment. The medium was then replaced by 400 μl of alloys extracts respectively. At the end of the incubation period, the medium were removed and the adherent cells were subjected to Live/Dead staining following the manufacturer’s protocol (Sigma, USA). Briefly, 1 μM calcien AM and 2 μM ethidium homodimer-1 solutions were prepared in PBS. After the removal of the extracts medium, the cells were washed with PBS for three times, followed by addition of 100 μL of 1 μM calcein AM and 2 μM ethidium homodimer-1 solution. Cells were then photographed using a fluorescence microscope. For quantitative analysis, the fluorescence intensity of the live cells which stained with green was calculated with the Image Pro^®^ software.

### Scratch assay

The scratch migration assay is a standard method for evaluating cell migration *in vitro*^59^. In our present study, it was adopted to evaluate the effect of the alloys extracts on the spreading and migration capabilities of the U2OS cells. U2OS cells were seeded in 12-well plates at a density of 5 × 10^5^ cells/ml in culture medium. Cells were cultured in a humid atmosphere with 5% CO_2_ at 37 °C until they had reached a confluence of about 90%. Then, consistently shaped straight scratches were made with a sterile 100-μl plastic pipette tip. Any cell debris was gently removed from the culture medium by washing with the culture medium. After the washing process, the cells were cultured with the alloys extracts for 24 h and then stained with 1 μM calcein AM. The migration of the cells across the scratched gap was assessed with a fluorescence microscope. The denuded area was calculated with ImagePro Plus 6.0 software.

### Cell invasion assay

Cell invasion assay was conducted by utilizing 24-well chambers with matrigel-coated filters (8 μm pore size). The Matrigel was diluted with serum-free medium with a ratio of 1:3 before usage. Subsequently, cells with a density of 10^5^ cell/ml were seeded into the membrane of the upper Matrigel chamber in 200 ul serum-free medium, and 600 μl medium containing 10% FBS was added to the lower chamber as an attractant. The chambers were then incubated in a humid atmosphere at 37 °C with 5% CO_2_ for 1 day to allow attachment. The upper medium was then replaced by 200 μl of extracts supplemented without FBS. After another 24 h incubation, cells on the opposite side of the membrane were stained with crystal violet (151000, Sigma) and photographed. This assay was performed in triplicate. For quantitative analysis, the number of cells invaded through the matrigel coated chamber were counted by the naked eyes. For each alloy extract, five random fields were selected, and the results were presented as mean ± std.

### U2OS cell cycle analysis

The distribution of cell cycles was determined using propidium iodide (PI, Sigma) staining by fluorescence-activated cell sorting (FACS) analysis. U2OS cells were digested with 0.25% trypsin and incubated in 25-cm^2^ culture flasks at a density of 1 × 10^5^ cells/ml in 4 ml medium for 24 h and starved for another 24 h in serum-free RIMP-1640 medium. After the starving procedure, the cells were then treated with the alloys extracts for another 48 h. Next, the cultured cells were harvested and washed in cold PBS, then the cells were fixed in 70% cold alcohol at 4 ^o^C overnight and washed with ice-cold PBS again before usage. Cells were then incubated with PI solution (40 mg/ml) for 30 min at room temperature in the dark. And cells were analyzed on a Guava EasyCyte 5HT flow cytometer. The data were analyzed by Guava Incyte Software v2.2.2.

### U2OS cells apoptosis analysis

The apoptotic studies were carried out using Guava Nexin Reagent (Millipore). Cells with different types such as normal cells, early apoptotic cells and late apoptotic cells can be distinguished by this apoptosis study using flow cytometer^60^. The Guava Nexin assay uses two stains (annexin V and 7-amino actinomycin D (7-AAD)) to quantitate the percentage of apoptotic cells. It was conducted according to the manufacturer’s protocol. In brief, after treatment with the alloys extracts for 24h, U2OS cells were collected and resuspended in 100ml medium supplemented with 100 μl 1% FBS. The cells were then incubated with 100 μl Annexin V-PE and 7-AAD labeling solution (Millipore) for 20 min at room temperature in the dark. Cells positive for AnV only (early apoptotic) or AnV and 7-AAD-positive cells (late apoptotic) were quantified by Guava EasyCyte 5HT flow cytometer. The data were analyzed by Guava Nexin Software v2.2.2.

### Western Blotting

Western blotting assay was applied to quantify related protein expression in different signal pathways. U2OS cells after incubated in the alloys extracts for 24 h were washed twice with ice-cold PBS and detached from the culture plate. The detached cells were then transferred into ice-cold RIPA lysis buffer. The cell lysates were collected and centrifuged at 15000 rpm for 15 min at 4 °C and the precipitates were discarded. The total protein concentration in the supernates was determined by BCA protein assay Kit (Peirce). For western blot analysis, after being heated for 5 min at 95 °C in a sample buffer, equal amounts of proteins from each sample (20 μg) were separated by 10% sodium dodecyl sulfate polyacrylamide gels (SDS-PAGE) via electrophoresis and transferred to PVDF membranes (Millipore). The membranes were blocked for 2 h with 3% bovine serum albumin. Membranes were firstly probed with primary antibodies (1:1000) against p53, Bax, Bcl-2, p38, JNK, ERK1/2, p-p38, p-JNK, p-ERK1/2, β-actin and GAPDH overnight at 4 °C and then incubated in horseradish peroxidase (HRP) -conjugated secondary antibodies (1:5000). After incubation, the membranes were washed 3–5 times with Tris-Buffered Saline and Tween 20 (TBST) buffer. Signals were then visualized by enhanced chemiluminescence detection reagents (Millipore) and developed with Kodak films.

### Statistical analysiss

The data was presented as mean ± standard . Statistical analysis was performed with GraphPad Prism® 5.01 software. Statistical analysis was conducted using the standard analysis of variance method and post-hoc analysis with the Bonferroni correction. A *p*-value <0.05 was considered statistically significant difference.

## Additional Information

**How to cite this article**: Wu, Y. *et al.* Unique antitumor property of the Mg-Ca-Sr alloys with addition of Zn. *Sci. Rep.* **6**, 21736; doi: 10.1038/srep21736 (2016).
